# Optimizing referral pathways to a community based organization for early‐stage Alzheimer’s and dementia Cognitive Stimulation Therapy services

**DOI:** 10.1002/alz.089921

**Published:** 2025-01-09

**Authors:** Stephani Shivers, Lauren Tiede, Annabess Ehrhardt, Edward Cisek, Deanna Curbeam, Michael Lepore

**Affiliations:** ^1^ CaringKind, New York, NY USA; ^2^ University of Massachusetts Amherst, Amherst, MA USA

## Abstract

Community‐based organizations (CBOs) are an essential part of dementia service delivery to people living with dementia (PLWD) and family caregivers (CG) impacted by Alzheimer’s and other dementias, however use of CBO services particularly by PLWD is limited. Referral of PLWD to CBOs by health systems (HS) and health care providers (HCPs) is a common way that PLWD come to use CBO services, but integration between CBOs and HS/HCPs is inconsistent. For example, referrals to CBOs might come from HS/HCPs or from other CBOs, these referrers might provide referrals for a single program or for multiple different programs, and the referrers might be physicians, social workers, case managers, or other clinicians. Aiming to advance effective integration with the broader constellation of HS/HCPs and CBOs, CaringKind, a CBO providing dementia services, examined the outcomes of referrals from different clinical contexts and different referral sources. This program analysis leverages CaringKind’s referral data and information on enrollment and participation in CaringKind’s Cognitive Stimulation Therapy (CST) program, which receives referrals as one of many programs available in standard operating procedures, and receives referrals in the context of a pragmatic trial with no other program options.

Drawing on referrals to CaringKind for CST over the course of an 18‐month period, we calculated the rates of conversion from CST referral to CST participant to count successful referrals. We then compared the successful referral rates in standard operations with the successful referral rates in the pragmatic trial. Results show the percentage of successful referrals from physicians in the pragmatic trial compared to providers in standard practice. Lessons learned on the referral pathway process including the impact of re‐education to identify the most appropriate patients for CST will be highlighted. Participant outcomes and completion rates gathered for program evaluation will also be shared.

